# Supplementary figures and images for: Constitutive polysaccharide degradation and diet-dependent lipid metabolism reveal an adaptive feeding strategy in the pacific oyster Magallana gigas
Source: PLoS One. 2026 Apr 29;21(4):e0347644. doi: 10.1371/journal.pone.0347644 (PMC13128122; doi:10.1371/journal.pone.0347644)

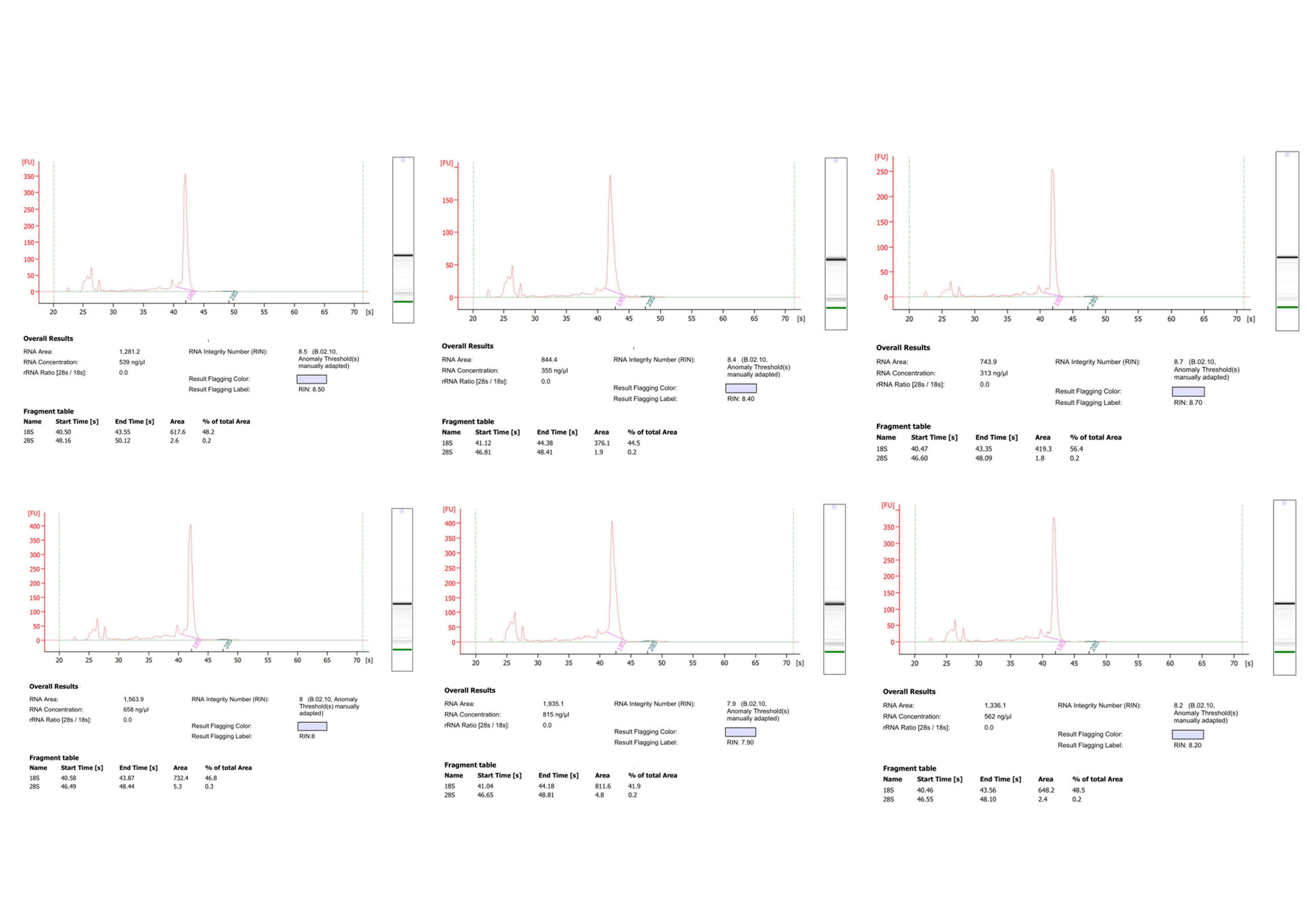

Supplement: S1 Fig — Top row, from left to right: reed leaf–fed group replicates 1–3. Bottom row, from left to right: diatom-fed group replicates 1–3. (TIF) [file pone.0347644.s001.tif]

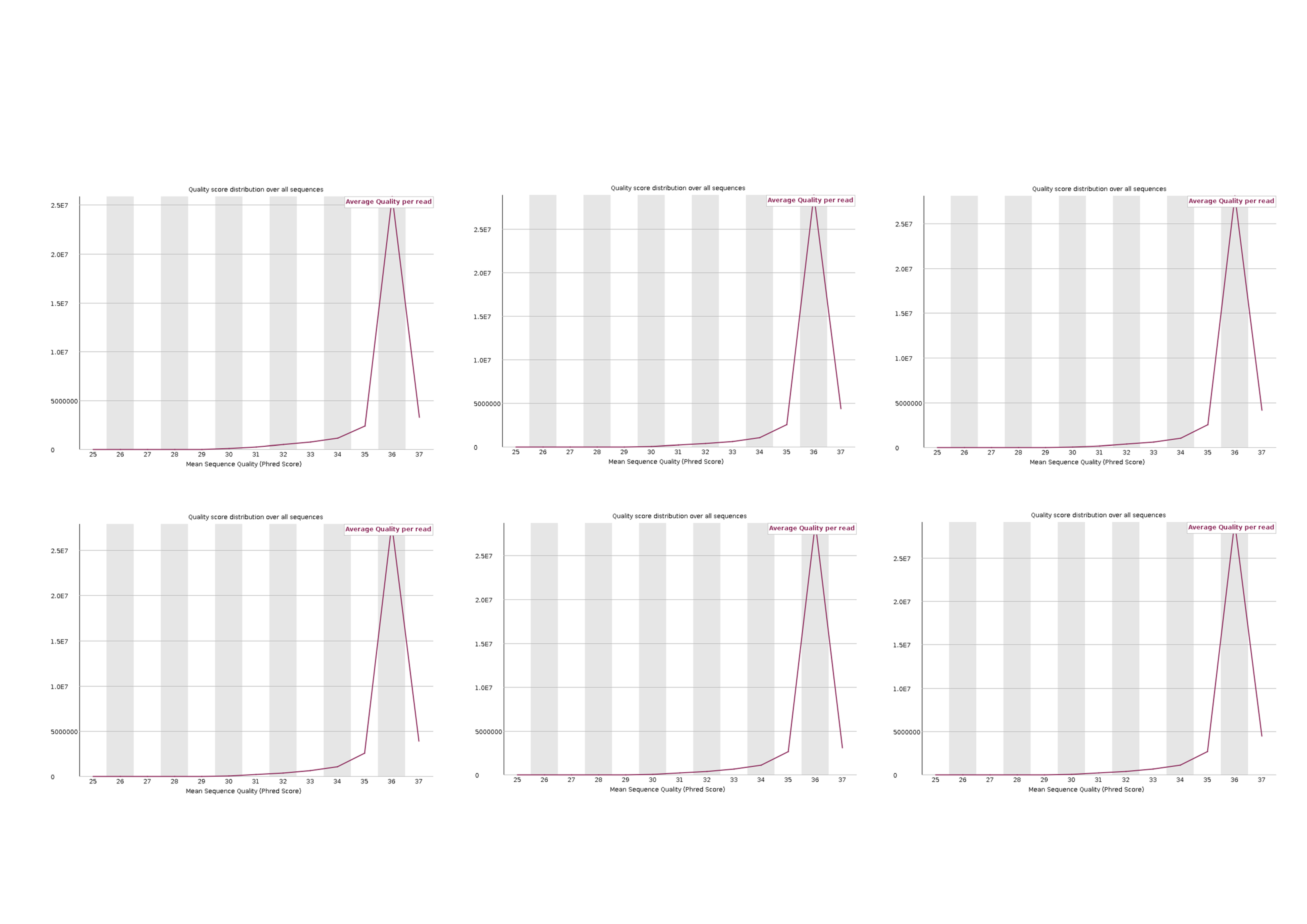

Supplement: S2 Fig — Top row, from left to right: reed leaf–fed group replicates 1–3. Bottom row, from left to right: diatom-fed group replicates 1–3. (TIF) [file pone.0347644.s002.tif]

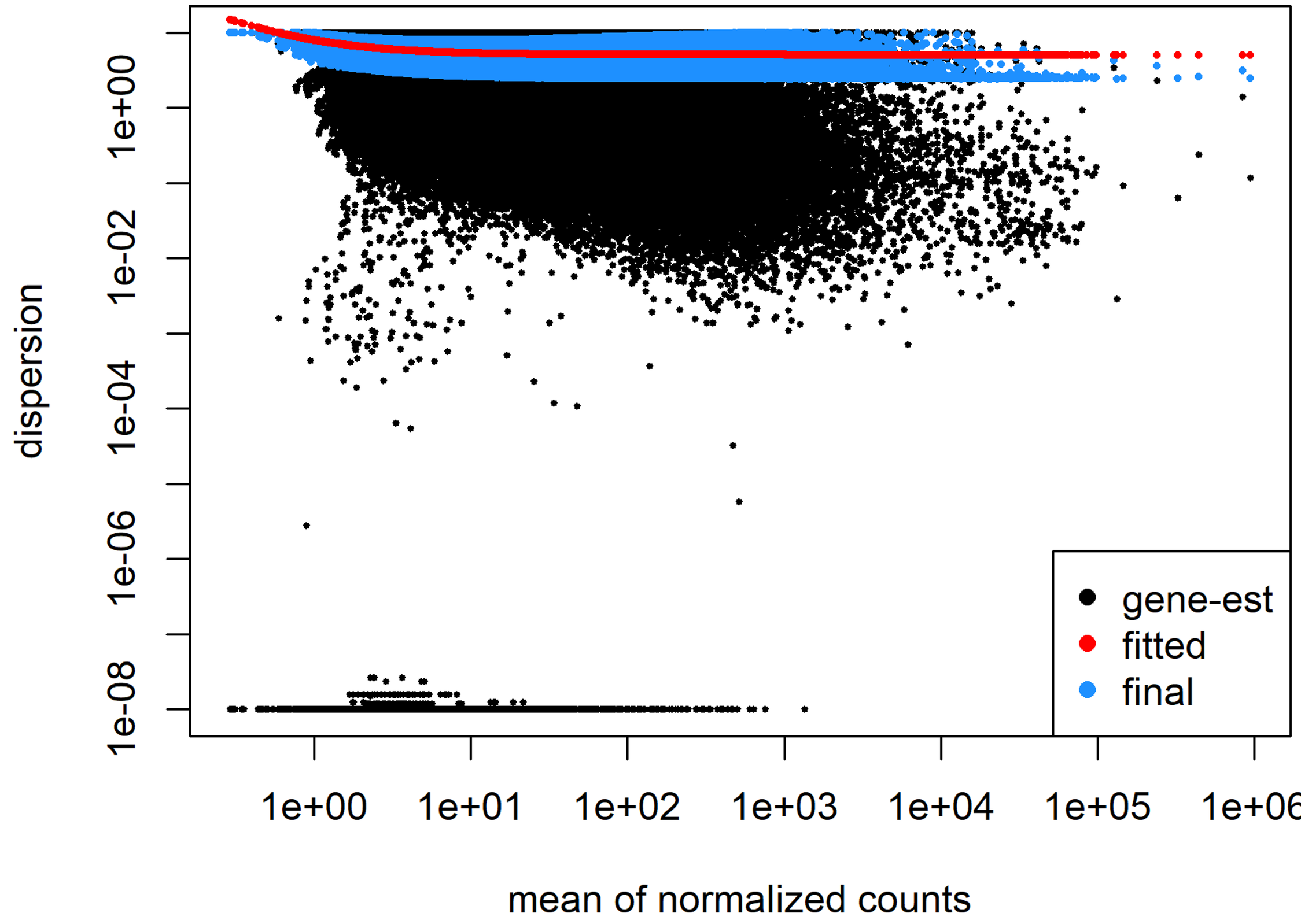

Supplement: S3 Fig — Each point represents an individual transcript. Black dots indicate gene-wise dispersion estimates, red dots represent the fitted dispersion trend, and blue dots show the final dispersion estimates after shrinkage. The final dispersion estimates (blue) closely follow the fitted trend (red), indicating appropriate modeling of the mean–variance relationship and reliable estimation of dispersion across transcripts. (TIF) [file pone.0347644.s003.tif]
